# Exploration of *Phyllanthus acidus* mediated silver nanoparticles and its activity against infectious bacterial pathogen

**DOI:** 10.1186/s13065-018-0412-7

**Published:** 2018-04-20

**Authors:** Cherukuri Sowmya, Vuppalapati Lavakumar, Narayanan Venkateshan, Velayutham Ravichandiran, D. V. R. Saigopal

**Affiliations:** 1 Drug Delivery and Nanotechnology Laboratory (DDNL), Department of Pharmaceutics, Arulmigu Kalasalingam College of Pharmacy, Anand Nagar, Krishnankoil, Virudhunagar Dist, Srivilliputtur, 626126 Tamil Nadu India; 2 National Institute of Pharamceutical Education and Research, NIPER - Kolkata at Indian Institute of Chemical Biology, Raja S. C. Mullick Road, Jadavpur, 700032 Kolkata India; 3 Department of Virology, Sri Venkateswara University, Tirupati, 517570 Andhra Pradesh India

**Keywords:** *Phyllanthus acidus*, Silver nanoparticles, Antibacterial activity, Fluorescence microscopy studies, Cell surface morphology studies

## Abstract

In our present investigation, synthesis of nontoxic, eco friendly and cost effective silver nanoparticles, *Phyllanthus acidus* (*P. acidus*) was used as starting material. The influence of phyto-constituents present in aqueous extracts of *Phyllanthus acidus* was found to be effective in reduction of silver nitrate to free silver nanoparticles (PA-AgNPs). HPTLC finger print analysis reveals the presence of flavonoid, quercetin in aqueous extracts of *Phyllanthus acidus*. Surface plasmon racemonance exhibited λ max at 462 nm through UV–Vis spectroscopy. Zeta size revealed that the size of nanoparticles were with in the range of 65–250 nm with polydisperse index (PDI) of 0.451. The negative charge of zeta potential value (− 16.4) indicates repulsion among PA-AgNPs with their excellent stability. FESEM-EDAX, XRD and TEM analysis confirmed the presence of nano-crystalline PA-AgNPs with different morphological textures. Further, PA-AgNPs has shown potent antibacterial effect on *E. coli* cells. The greater antibacterial effect (viable and dead cells) of PA-AgNPs were confirmed by using acridine orange (AO) dye which can able to provide insight of healthy as well as damaged DNA. Live cells emit florescence green and dead cells (treated with PA-AgNPS at 20 and 40 µg/ml) appear as pale orange red colour. Post treatment, investigations of PA-AgNPs on *E. coli* cells under SEM was found to be effective against cell membrane damages which leads to cell death or cell growth arrest. Hence, from the above findings, we strongly recommend silver nanoparticles from *Phyllanthus acidus* can be used as a potential source for antimicrobial agent for chronic infections and also against other harmful microorganisms.
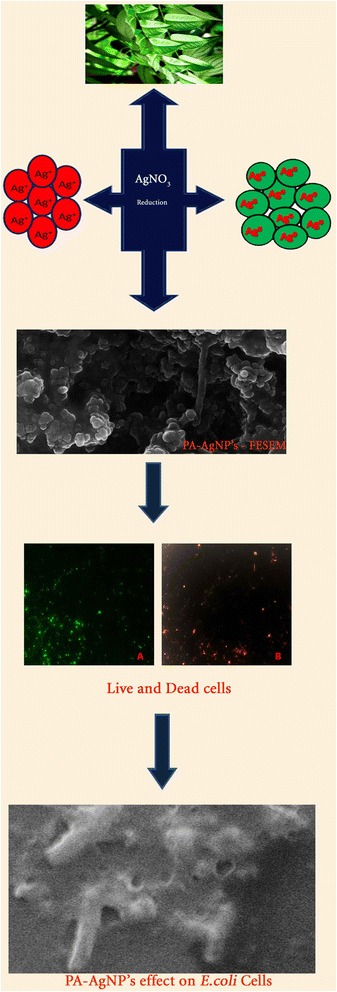

## Introduction

For years together, microbes are complicated and it was difficult to restrict their growth or to prevent its infection towards human beings. The current accessible drug regimen on prolonged treatment was making these microbial pathogens to become resistant and causing urge in need of newer anti microbial agents with superior mode of action [1, 2]. In recent days, nanotechnology and nano scale materials have emerged as potent delivery materials for many ailments [3]. This may be due to its size, unique surface area to volume ratio and chemical complexities has created a new platform to fight against many diseases. Among promising nano materials, metallic silver nanoparticles plays a pivotal role in exhibiting antibacterial property [4]. Many studies support that nanonization of silver found to be effective against various microbial pathogens like *Staphylococcus aureus*, *Escherichia coli*, *Vibrio cholerae*, *Pseudomonas aeruginosa* and *Salmonella typhi* [5, 6]. The recent available methods of fabrication of metallic silver nanoparticles are capital intensive, toxic, non eco-friendly and have low productivity leading towards the hunt for alternative and high capacitive nanoparticles. The biosynthesized routes otherwise called as “Green synthesis” methods were under wide exploration due to their amenability towards better biological functionalizations. The formation of silver nanoparticles were based on the type of solvent and nature of reducing agents (biomolecules) which acts as capping agent in the formation of free silver ions [6]. Such silver nanoparticles have wide range of application in various disciplines like (a) biological tags and biosensors in diagnostic applications, (b) incorporating Ag-NPs in apparel, footwear, wound dressings, paints, cosmetics and plastics resulted in antibacterial properties, (c) Ag-NPs as conductive inks in composites as enhanced thermal and electrical conductivity and (d) Ag-NPs exhibit as metal-enhanced fluorescence (MEF) and surface-enhanced Raman scattering (SERS) thus leading to optical applications.‘

Hence, our investigation was focused on fabrication of eco friendly silver nanoparticles by using green source, such as aqueous leaf extracts of *Phyllanthus acidus* (*P. acidus*) which had significant economic importance. However, *Phyllanthus acidus* was said to be having wide range of phytoconstituents including terpenoids, alkaloids, flavonoids and tannins, which have been shown to posse’s useful biological activity in in vitro and in vivo. Moreover, *P. acidus* claims as anti inflammatory, anticancer, antiplatelet, antipyretic, antiviral, antimutagenic, antiallergic and antibacterial. Here, we have reported a novel method for synthesis of silver nanoparticles from the leaves of *P. acidus* and its antimicrobial potential against *E. coli* cells.

## Materials and methods

### Materials

All chemicals used in this study were obtained from Sigma (Bangalore, India) and Merck (Mumbai, India). *Phyllanthus acidus* leaves were collected from the local area of Srivilliputtur and authenticated by Dr. Madav Shetty, Professor, Department of Botany, S.V. University, Tirupati. Voucher specimen (AKCP-PH-0254) was deposited in the Pharmacognosy Laboratory, Arulmigu Kalasalingam College of Pharmacy, Krishnankoil, India.

### Preparation of *P. acidus* extract

Fresh leaves of *P. acidus* (200 g) were collected in the month of February. The leaves were brought to DDN lab and washed with double distilled water to get rid of dust particulates present on them. The leaves were reduced to tiny size and shade dried for 1 week. The material was powdered (Coarse) by using mixer grinder and then sieved to get fine particles. About 100 g of powder was suspended in 400 ml of double distilled water, heated at 65 °C for 60 min, filtered through Watman filter paper No. 2 and the filtrate was stored in the refrigerator at − 4 °C for further experimental purpose.

### Preliminary phytochemical screening and HPTLC finger print analysis

Preliminary phytochemical analysis for *P. acidus* extracts were carried out as per the protocol mentioned in Harbore, 1998 [7]. HPTLC (silica gel G 60F254 TLC plates of E. Merck, layer thickness 0.2 mm) fingerprint analysis was established for aqueous extracts of *P. acidus*. HPTLC was performed on (10 cm × 10 cm) aluminum backed plates coated with silica gel 60F254 (Merck, Mumbai, India). Standard solution of quercetin and test were applied on the plates as bands of 8.0 mm wide, 30.0 mm apart, and 10.0 mm from the bottom edge of the same chromatographic plate by use of Camag (Muttenz, Switzerland) Linomat V sample applicator equipped with 100 µl Hamilton (USA) syringe. Ascending development to a distance of 80 mm was performed at room temperature (28 ± 2 °C), with toluene:ethyl acetate:formic acid [(5:4:1) (v/v/v)], as mobile phase in a Camag glass twin trough chamber previously saturated with mobile phase vapour for 20 min, Quercetin of (100 µg/ml) was used as standard [8].

### Synthesis of *Phyllanthus acidus* (PA-AgNPs) silver nanoparticles

Aqueous solution (1 mM) of silver nitrate (AgNO_3_; mol. wt: 169.87) was prepared and used for the synthesis of *P. acidus* silver nanoparticles (PA-AgNPs). Pipette out 400 µl of extracts (previously extracted and stored), added to 40 ml of 1 mM AgNO_3_ solution and kept in shaker for 10 min to promote the reduction. The reaction mixture was set to continue for 24 h in the dark at a temperature of (25 ± 2 °C). The silver nanoparticles thus obtained were purified by repeated centrifugation at 10,000 rpm for 10 min followed by redispersion in milliQ water [9].

### Characterization studies

The absorption spectra for synthesized silver nanoparticles (PA-AgNPs) were measured by using a Shimadzu spectrophotometer (UV-1700) at 300–800 nm range. Particle size and zeta potential were assessed by using HORIBA Instruments (Singapore) Pvt Ltd, Singapore [10]. For X-ray diffraction (XRD) analysis, PA-AgNPs sample was spread on thin aluminium foil by dropping 100 µl of sample, dried at 50 °C and subjected into Rigaku smart lab instrument, operated at a voltage of 40 kV 30 mA current having Cu Kα1 radiations [11]. Size and elemental composition was confirmed by using Field emission scanning electron microscopy-energy dispersive X-ray analysis (FESEM-EDAX) by SUPRA 55-CARL ZEISS, Germany. The morphology and size were further confirmed by Transverse electron microscopy (TEM) by coating onto carbon coated grids performed by JEOL 3010 instrument, operated at an accelerating voltage of 300 kV.

### Antibacterial activity of PA-AgNPs against *E. coli by* agar well diffusion techinique

For antibacterial evaluation of PA-AgNPs, *Escherichia coli* (*MTCC 443*) was selected as target organism. Prior to experimentation, *E. coli* cells were subcultured by using nutrient broth and Muller Hinton Agar (MHA) plates were prepared for antibacterial assay. Bacterial suspension was swabbed on MHA plates. Three wells (6 mm diameter) were pierced and test samples (10 and 20 µg/ml of PA-AgNPs) and standard (15 µg/ml of streptomycin sulfate) were seeded in to the wells and zone of inhibition was measured after 18 h of  incubation [12].

### Effect of PA-AgNPs on *E. coli* cell surface morphology

The change in surface morphology of *E. coli* was examined after treating with PA-AgNPs at 20 µg/ml [13]. Post treatment, samples were kept in a shaker incubator at 150 rpm for 6 h. After treatment, a small drop of culture was fixed on glass grids (8 mm × 8 mm) with micro pipette, treated with graded methanol (to eliminate moisture in bacterial cell) with a method of critical-point drying [14]. The same procedure was repeated for untreated cells. Morphological changes in *E. coli* was examined using scanning electron microscope (SEM) JEOL-JSM-6060 model at 15–20 kV range.

### Live and dead cells assay

Live and dead cell assessment was carried out for PA-AgNPs by using acridine orange (HiMedia laboratories, India) as staining reagent [15]. The stock was prepared by mixing of 0.05 ml of 1% Acridine orange with 5 ml of acetate buffer with 0.2 M (pH 4.0). Bacterial sample (*E. coli*) was spread on sterile glass slide, dried at 50 °C, fixed in absolute methanol for 2 min and air dried. The slides were then covered with acridine orange (AO) for 1 min, gently rinsed with tap water and air dried [16]. The samples were examined with epifluorescence microscope at 460–490 nm excitation (Eclipse 80i, Nikon, Japan).

## Results and discussion

### Preliminary phytochemical screening and HPTLC finger print analysis

**Fig. 1:**
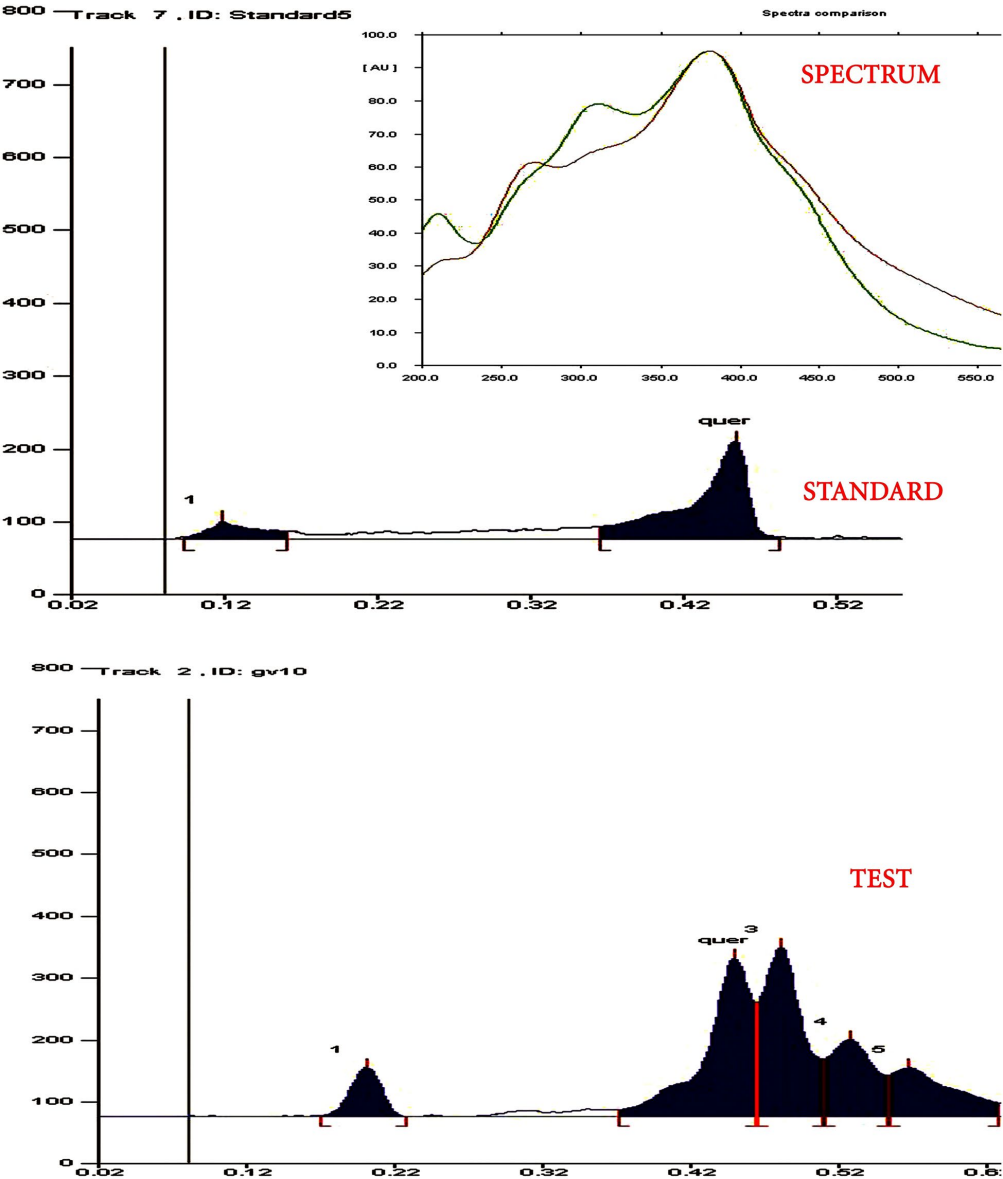
HPTLC finger print analysis of *P. acidus* extracts

Preliminary phytochemical investigation divulges the presence of glycosides, phenolic compounds, flavonoids, proteins, amino acids, carbohydrates and saponins in aqueous extracts of *P. acidus*. Hence, *P. acidus* extract containing higher altitude of phytoconstituents which may possibly take part in reactions in effective reduction of silver to free silver ions [17]. However, HPTLC finger print analysis also confirms the presence of quercetin (Fig. 1) flavonoid which has influenced the conversion of Ag^+^ to Ag^0^ ions due to easily oxidizable conjugated hydroxyl groups in the molecule [18].

### Synthesis of *Phyllanthus acidus* (PA-AgNPs) silver nanoparticles

**Fig. 2:**
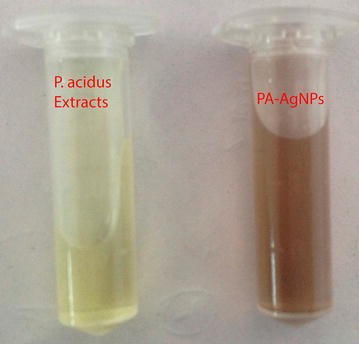
Preliminary conformation of formation of PA-AgNPs

Synthesis of silver nanoparticles were carried out in the presence of aqueous leaf extracts of *Phyllanthus acidus.* The color change was very rapid after addition of *P. acidus* extracts which turns the solution into brown within 10 min and turned to dark brown after 2 h. Color change from pale yellow to dark brown (Fig. 2) remained constant even after 24 h, which indicates the preliminary confirmation of formation of silver nanoparticles. It may be due to the reduction of silver nitrate into free silver nanoparticles by the influence of various conjugated hydroxyl groups present in the *P. acidus* extracts [19].

### Characterization studies

**Fig. 3:**
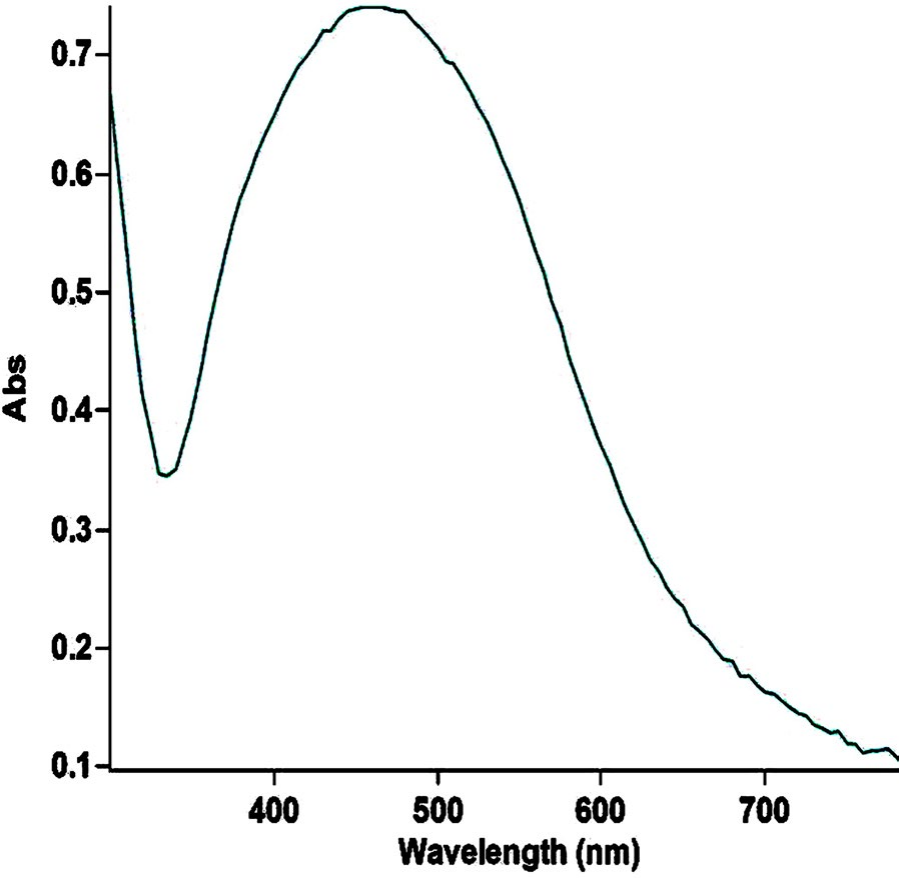
UV–Vis spectrum of PA–AgNPs mediated through *Phyllanthus acidus*

**Fig. 4:**
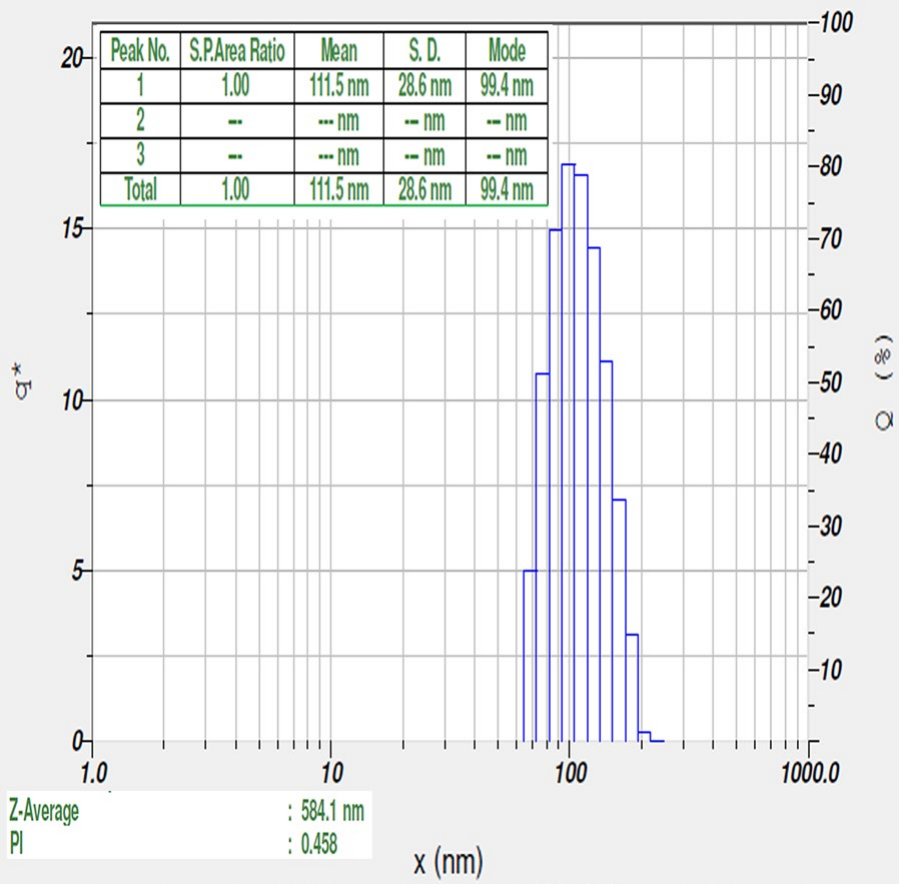
Particle size distribution of PA-AgNPs

**Fig. 5:**
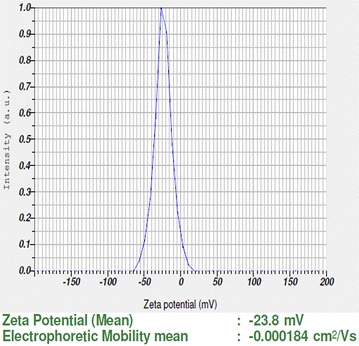
Zeta potential of synthesized PA-AgNPs

**Fig. 6:**
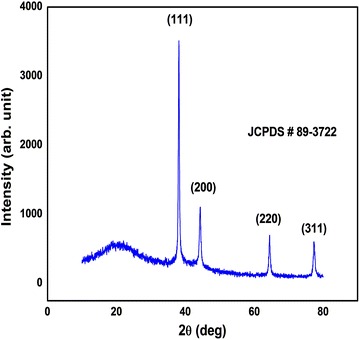
X-ray diffraction (XRD) pattern showing peaks for PA-AgNPs

**Fig. 7:**
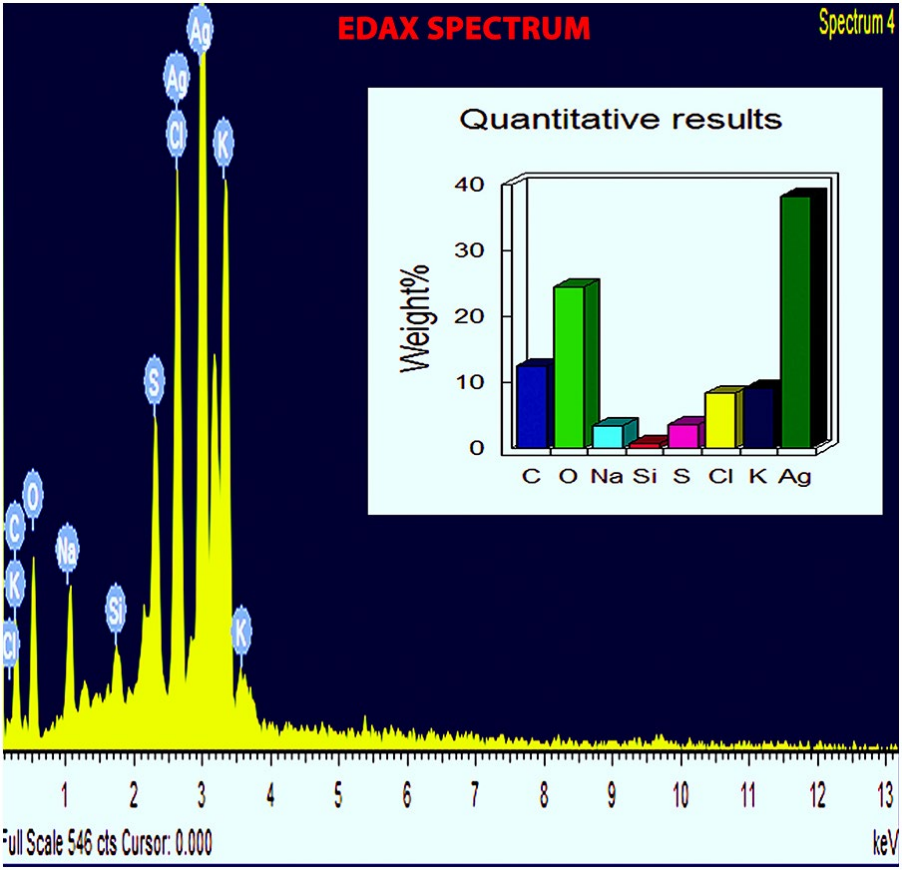
EDAX profiles of synthesized PA-AgNPs

**Fig. 8:**
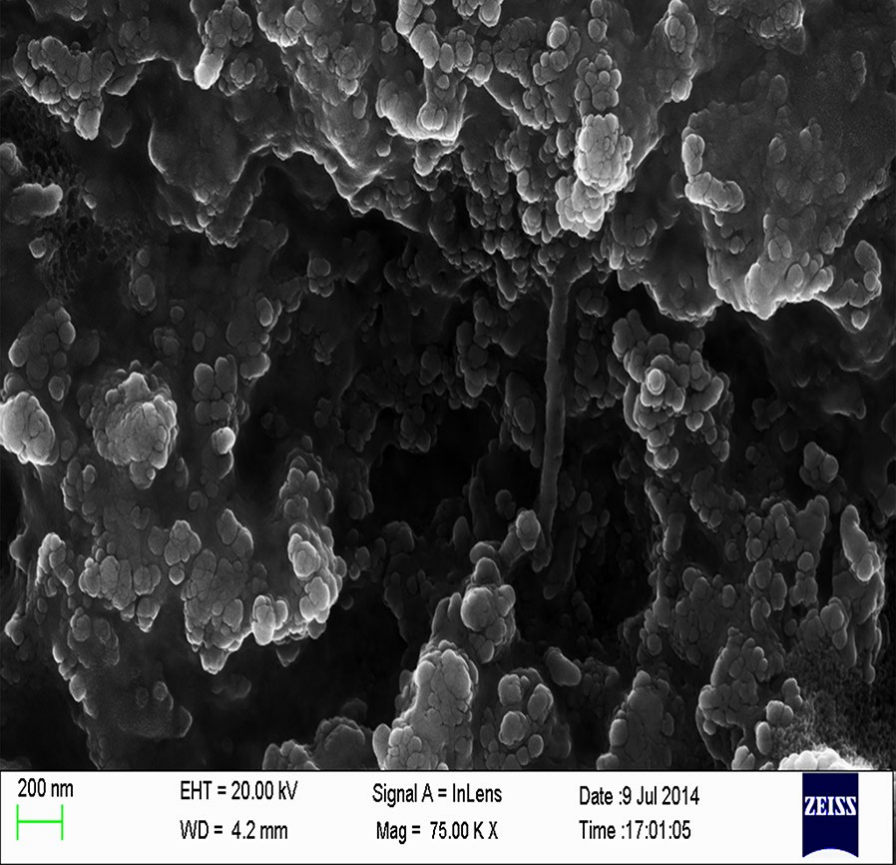
Scanning electron microscopy shows the various size ranges of formed silver nanoparticles (PA-AgNPs)

**Fig. 9:**
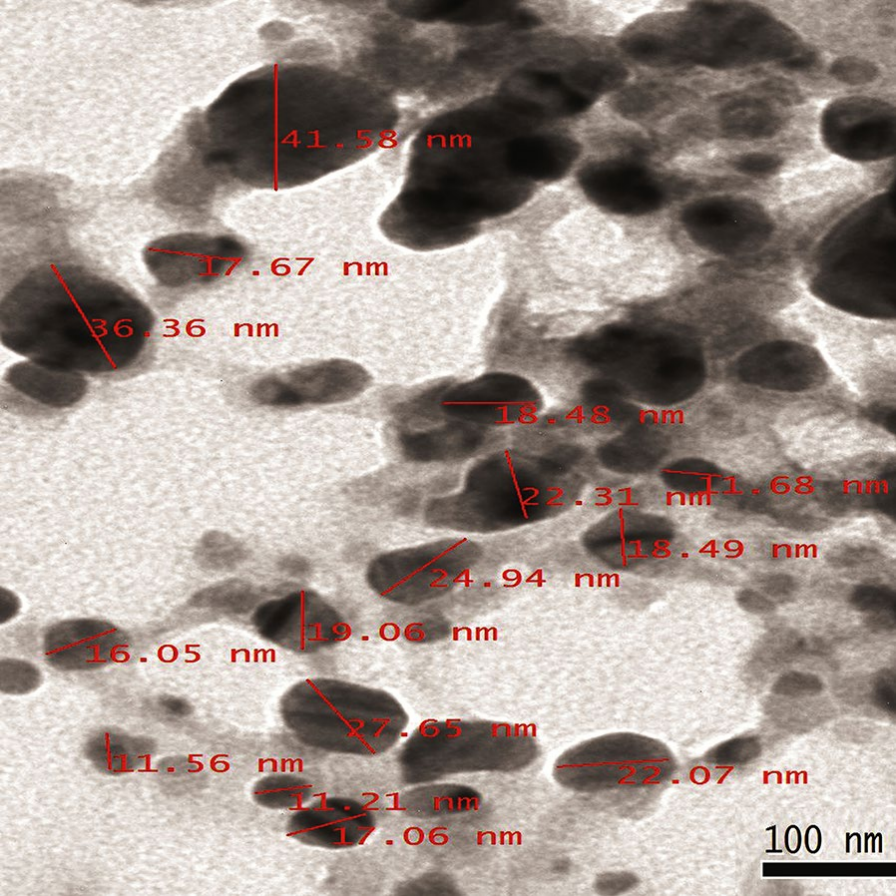
TEM micrograph image of bio-reduced silver nanoparticles (PA-AgNPs)

UV–Vis spectra of synthesized PA-AgNPs (Fig. 3) reveals the sharp intensity peak at 462 nm which was assumed to correspond to the silver nanoparticles. This excitation is due to induced electromagnetic field among nanoparticles by collective oscillation and conduction of electrons [20]. Particle size (Fig. 4) determination of synthesized PA-AgNPs were shown under by intensity. Laser diffraction reveals that particles obtained are in polydisperse (PI − 0.458**)** with the size range of 65–250 nm. Zeta potential of the synthesized (Fig. 5) PA-AgNPs was found to be − 23.8. The negative amplification by surface charge among nanoparticles could increase the electrostatic repulsion and repress the agglomeration which retains the particles as highly stable [21]. XRD is a commonly used method to determine the crystal structures of nanoparticles. X-ray diffraction (XRD) pattern of PA-AgNPs (Fig. 6) were compared with standard XRD files of PA-AgNPs published by the Joint Committee on Powder Diffraction Standards (JCPDS file no. 89-3722). Intense peaks occurred at 2θ peak values at 38.11°, 44.23°, 64.43° and 77.49° corresponds to (111), (200), (220) and (311) set of lattice planes respectively [22]. The pattern of silver nano particles were further confirmed by FESEM-EDAX (Fig. 7). The size (diameter) was clearly distinguishable at 200 nm range. The outcome of FESEM information (Fig. 8) was overlayed with earlier reports [23, 24]. The strong signal peak of silver in the synthesized product was due to surface plasmon racemonance, confirmed by EDAX analysis at 3 keV. Moreover, weaker signals from oxygen and other atoms were observed and this may be due to the bounce of biomolecules on to the surface of synthesized PA-AgNPs [25]. TEM studies reveals exact shape and size of the PA-AgNPs. Figure 9 shows spherical and polydispersed silver nanoparticles formed by using aqueous extracts of *Phyllanthus acidus* leaves. It is quite interesting that these nanoparticles were encapsulated/bounded with organic biomolecules from the leaf extract [26].

### Antibacterial activity of PA-AgNPs against *E. coli by* agar well diffusion techinique

**Fig. 10:**
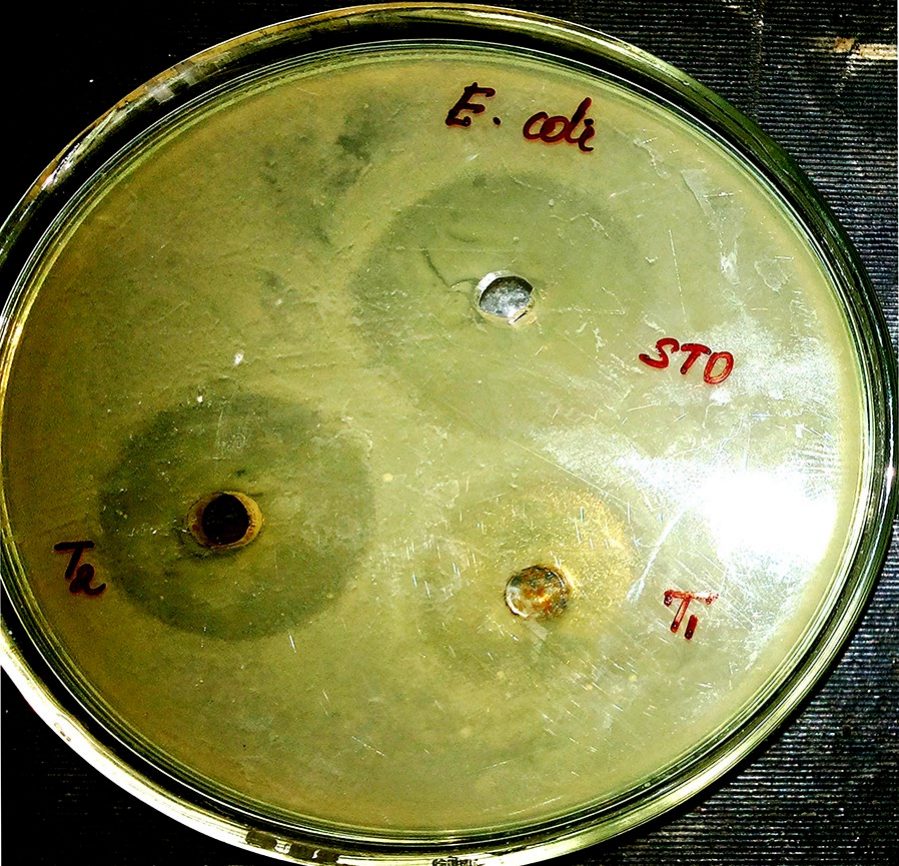
Antibacterial activity of PA-AgNPs against gram negative bacteria (*E. coli*). Test (T1—10 µg/ml; T2—20 µg/ml; STD—15 µg/ml)

Antibacterial assay was carried out in triplicates by agar well diffusion technique, which is a regular technique used to estimate the sensitivity of microbial strains against a wide range of antibiotics at different concentrations in order to prove as potent antimicrobial agent. Figure 10 reveals that the antibacterial effect of PA-AgNPs against gram negative bacteria in terms of zone of inhibition at 10 µg/ml (24 mm) and 20 µg/ml (36 mm) which were compared to that of standard streptomycin sulphate at concentration of 15 µg/ml (39 mm). The possible mechanism behind formation of clear zones were due to the interference of silver nanoparticles on the bacterial cell proliferation [27].

### Effect of PA-AgNPs on *E. coli* cell surface morphology

**Fig. 11:**
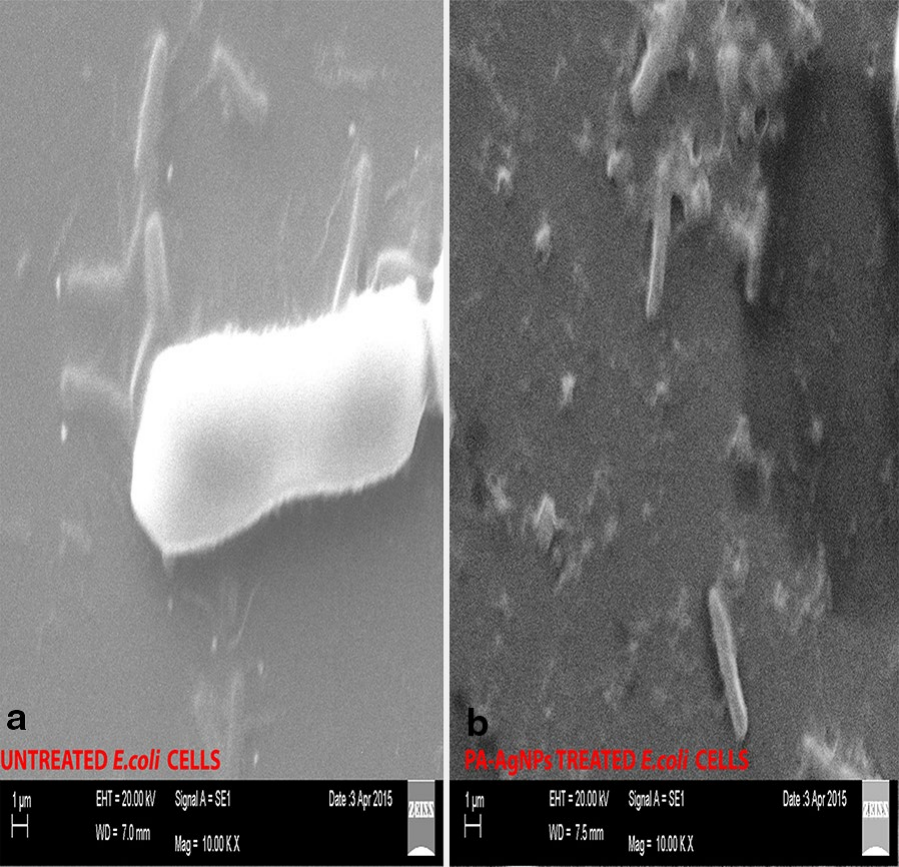
Morphological changes of *E. coli* cells under scanning electron microscopy before (**a**) and after (**b**) treatment with PA-AgNPS at 20 µg/ml

The cell surface morphology of *E. coli* was extensively studied by using scanning electron microscopy. Post treatment with PA-AgNPs at the concentration of 20 µg/ml has shown the significant effect on the *E. coli* cell membrane which was compared with control. Figure 11b exhibits the formation of rumples, large surface collapses, abnormal cell breaking and complete lysis or dead cell formation in PA-AgNPs treated cells, whereas such kind of impressions were completely absent in untreated *E. coli* cells (Fig. 11a). Surface collapse and other abnormalities in *E. coli* was due to direct interference of PA-AgNPs on cellular biochemical process by pertaining interactions with thiol and amino groups in proteins associated with nucleic acids within cell membranes [28, 29]. This disruption of the cell wall is by direct production of increased levels of reactive oxygen species (ROS), mostly hydroxyl radicals and singlet oxygen causing interruption of cellular functions and leads to disorganization of membrane [30, 31]. However, the above said fact may be high in case of *E. coli* due to surface collapse of the cell wall which is less dense and it easily facilitates the inward flow of PA-AgNPs. Futher, these PA-AgNPs cause distinct damage to respiratory chain dehydrogenases, thus leading to cell cycle arrest or cell death [6, 32].

### Live and dead cell assay

**Fig. 12:**
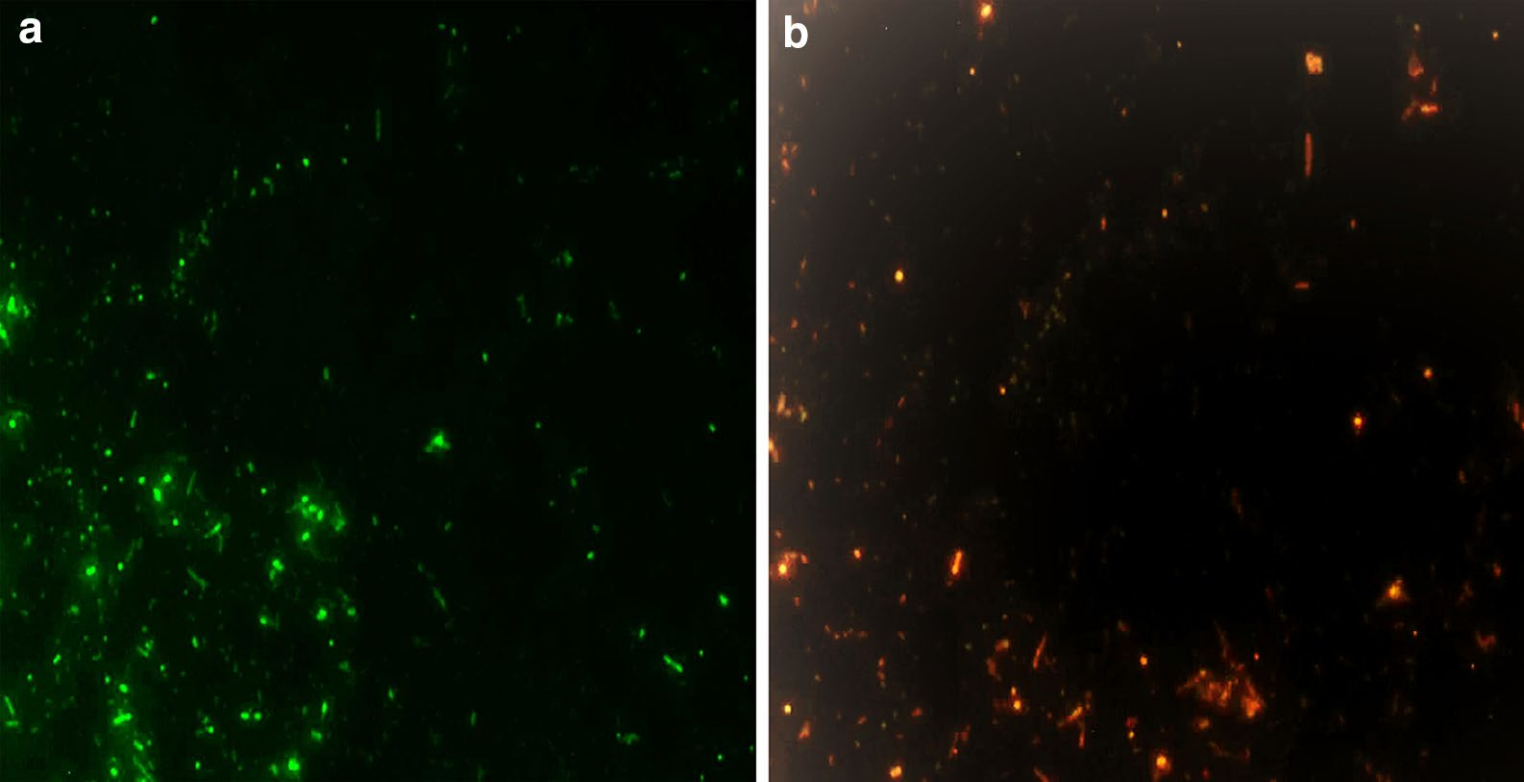
Fluorescence images of *E. coli* cells, **a** live cells and **b** dead cells

Bacterial assay (live and dead cell) was performed by using gram negative *E. coli* strains after 6 h post treatment with synthesized silver nanoparticles using Acridine orange (AO) as staining dye. The bacteria without addition of PA-AgNPs served as the control [33]. Under florescence microscope, viable cells appear as green whereas, nonviable cells appears as pale orange red color [34]. This green emittance under fluorescence microscope was due to passage of acridine orange (AO) dye through cell membrane and uptake of AO by internal cellular organelles. Figure 12a elicits florescence green colour at 460–490 nm due to electrostatic interaction of cationic dye deposition of AO on cell organelles [35]. Whereas, Fig. 12b emits pale orange red color by the direct influence of PA-AgNPs on intercalating regions of DNA, which breaks of ds DNA into ss RNA or ss DNA by that cells were dead and lost its potency in uptake of AO [36]. Hence, it reflects with pale orange red colors under florescence microscopy due to the presence of unwinded ds DNA [37].

## Conclusion

The present investigation enlightens the fabrication of simple, clean, efficient, cost effective and eco friendly stable silver nanoparticles from aqueous leaf extracts of *Phyllanthus acidus.* Whereas, *P. acidus* extracts were effectively broke down the Ag^+^ ions into Ag^0^ in short time. The synthesized nanoparticles were characterized by using UV–Vis spectrum, Zeta size and potential, XRD, FESEM-EDAX and TEM. Further, the PA-AgNPs were said to be having promising effect on gram −ve bacteria, such as *E. coli* by proving excellent antibacterial activity on cell growth inhibition. Hence, this research concludes that *Phyllanthus acidus* mediated nanoparticles can be used against wide range of bacterial pathogens and it could be the pavement for newer formulations in near future for large scale applicator in the field of drug discovery.
